# High-throughput sequencing yields the complete plastid genome of the endemic species *Phragmipedium kovachii* (Orchidaceae) from northeastern Peru

**DOI:** 10.1080/23802359.2024.2397979

**Published:** 2024-09-02

**Authors:** Jois V. Carrion, Jhordy Perez, Daniel Tineo, Martha S. Calderon, Ligia Garcia, Manuel Oliva, Oscar Gamarra Torres, Danilo E. Bustamante

**Affiliations:** a Instituto de Investigación para el Desarrollo Sustentable de Ceja de Selva (INDES-CES), Universidad Nacional Toribio Rodríguez de Mendoza, Chachapoyas, Peru; b Instituto de Investigación en Ingeniería Ambiental (INAM), Facultad de Ingeniería Civil y Ambiental (FICIAM), Universidad Nacional Toribio Rodríguez de Mendoza, Chachapoyas, Peru

**Keywords:** Biodiversity, endemic species, genome assembly, Peru, *Phragmipedium*

## Abstract

*Phragmipedium kovachii* is a species of orchid endemic to the Amazonas and San Martín regions. Unfortunately, its excessive extraction has made it a critically endangered species. In this study, we performed next-generation sequencing of *P. kovachii* (GenBank accession number OR348669) and assembled its complete chloroplast genome. The complete chloroplast genome of *P. kovachii* is A + T-rich (64.3%), measuring 152,918 bp in length. This plastid genome contains a total of 124 genes (77 protein-coding genes, 39 tRNAs, and eight rRNAs) and five pseudogenes, including a pair of inverted repeats (IRs) 25,116 bp in size and separated by a large single-copy (LSC) region of 89,216 bp and a small single-copy (SSC) region of 13,470 bp. This genome has a typical quadripartite organization following the structure of other Orchidaceae plastomes. Phylogenetic analyses revealed the close relationship between *P. kovachii* and *P. besseae.* This study contributes to the understanding of the phylogenetic relationships of the monophyletic group Cypripedioideae.

## Introduction

Orchidaceae is one of the largest families of flowering plants, representing approximately 10% of seed plants (Roberts and Dixon 2008; Wang et al. 2019). One of the most representative groups of this family is the monophyletic subfamily Cypripedioideae, commonly known as slipper orchids, which are characterized by flowers with a lip and a column (Cameron et al. 1999; Chase et al. 2003; Yen et al. 2022). This group is composed of approximately 200 species distributed in the five genera *Cypripedium*, *Mexipedium*, *Paphiopedilum*, *Phragmipedium*, and *Selenipedium* (Guo et al. 2012; Unruh et al. 2018). An orchid with one of the most beautiful flowers in the world is *Phragmipedium kovachii*, which is 10–20 cm in size with pink to purple petals and large lips up to 7.5 cm long and 4 cm wide (Atwood et al. 2002; Cribb 2007). *P. kovachii* is an endemic species of northeastern Peru restricted to the Amazonas and San Martín regions (Millán et al. 2007).

This species was first described as *P. kovachii* by Atwood et al. (2002) based on material that was illegally introduced to the US and deposited in the Selby Botanical Gardens. A few days later, this species was described as *Phragmipedium peruvianum* by Christenson (2002). The oldest name given to a plant is the correct name according to the norms of priority of the International Code of Botanical Nomenclature, with later names being relegated to synonymy (Turland et al. 2018). Accordingly, *P. peruvianum* is now a synonym of *P. kovachii* (Cribb 2007). The discovery of *P. kovachii* led to uncontrolled extraction of many specimens, in turn reducing its population size and distribution (Millán et al. 2007). Currently, *P. kovachii* is classified as critically endangered according to the Red List criteria (IUCN criterion and guideline) since its geographical distribution is less than 100 km^2^, while its population is restricted to 25 individuals (Cribb 2007). This will likely result in its extinction in the wild in the next 10 years (Rankou 2016).

In Peru, 11 species of the genus *Phragmipedium* are recognized, and *P. kovachii* is currently categorized as a threatened species according to Supreme Decree N°043-2006-AG (SERFOR 2020). Additionally, *P. kovachii* is a protected species according to Appendix I of the Convention on International Trade in Endangered Species of Wild Fauna and Flora (CITES 2017). This protection is on the basis of the IUCN criterion and guideline. Most studies on *P. kovachii* are limited to morphological analyses (Ruiz Sánchez 2021), and studies at the molecular level are insufficient (Leitch et al. 2009). Genetic and genomic analyses provide information about species gene transfer, cloning, domestication, and evolutionary information (Vu et al. 2020; Song et al. 2022). Accordingly, the aim of this study was to decodify the plastid genome of the critically endangered *P. kovachii* using next-generation sequencing technology in order to (i) give insights about plastome composition and (ii) better understand the evolutionary history of the species.

## Methodology

**Figure 1.**
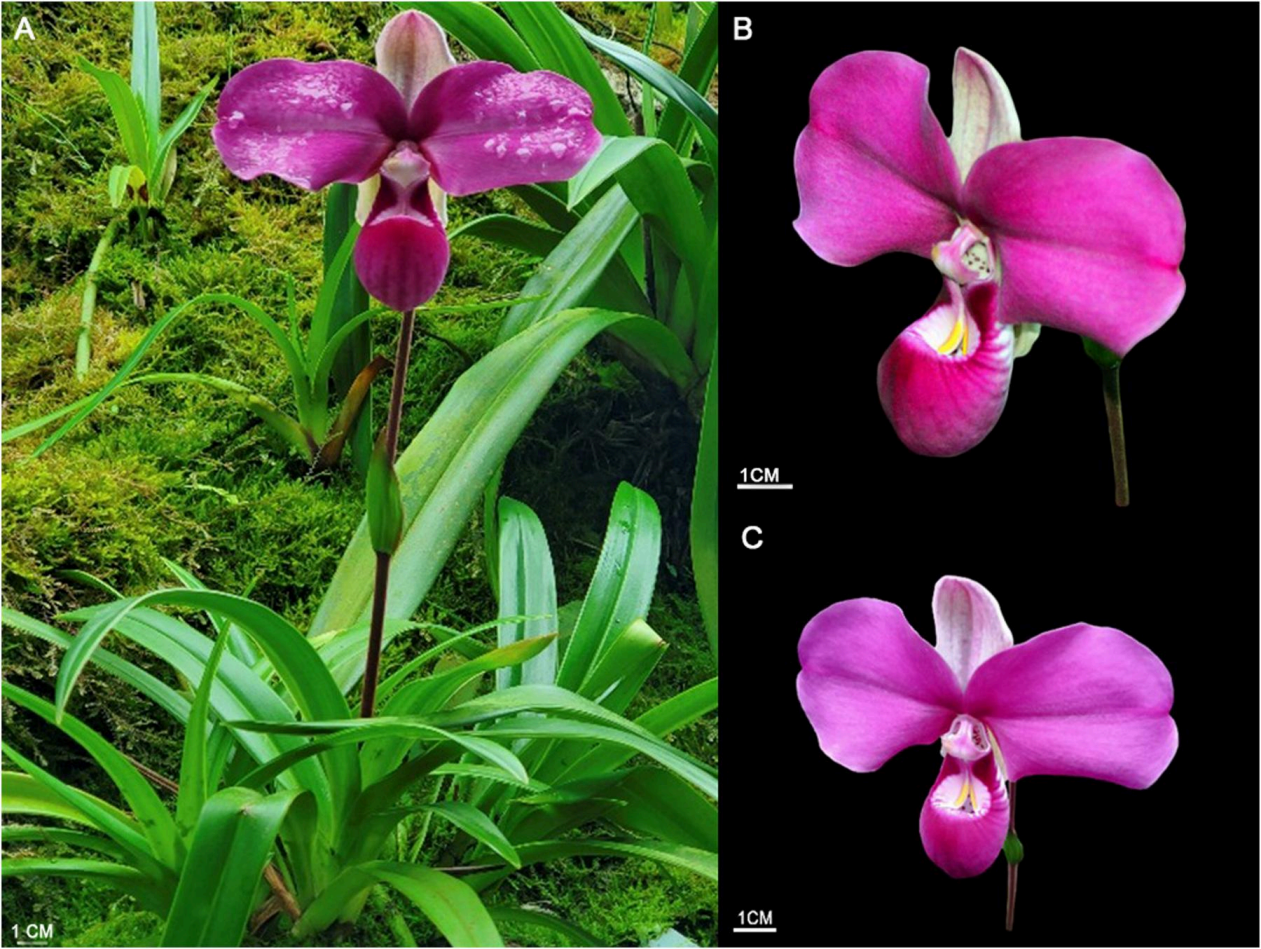
Morphology of *Phragmipedium kovachii* (KUELAP988). (A) Habit, showing a plant with a single flower. (B, C) Prominent flower with petals and a lip. All images were obtained from Yoiner Kalin Lapiz from the plant nursery Kgory Thika, Yambrasbamba, Bongará, Amazonas.

The specimen of *P. kovachii* was obtained by Jhon Charles Valle from the plant nursery Kgory Thika, Yambrasbamba, Bongará, Amazonas (5° 46′ 13.626″ S, 77° 53′ 55.409″ W). This specimen was an adult flowering plant 50 cm in height (Figure 1). Tissue samples of approximately 30 mm^2^ were taken from leaf tips for genomic analyses and placed in prelabeled 1.5 mL Safelock Eppendorf tubes added silica gel. This specimen was deposited in the herbarium of the Universidad Nacional Toribio Rodríguez de Mendoza (KUELAP, https://www.untrm.edu.pe, Curator Eli Pariente, email: eli.pariente@untrm.edu.pe) under voucher number KUELAP988.

Genomic DNA was extracted from *P. kovachii* (specimen voucher KUELAP988) using a NucleoSpin Kit (Macherey-Nagel, Düren, Germany) following the manufacturer’s instructions. The DNA was fragmented and ligated to unique adapters with the Swift 2S Turbo DNA Library Prep Kit (Swift Bioscience, Ann Arbor, MI). The 150 bp PE Illumina library was constructed and sequenced using the NovaSeq platform from Macrogen (Seoul, South Korea). The genome was assembled using default *de novo* settings in MEGAHIT (Li et al. 2021) and Geneious Prime 2023.2 (https://www.geneious.com) to close gaps. Sequencing depth and coverage were calculated following Yang et al. (2023). Genes were manually annotated using blastx, NCBI ORFfinder, and tRNAscan-SE 2.0 (Lowe and Chan 2016). The plastid genome of *P. kovachii* was aligned with other plastomes using MAFFT (Katoh and Standley 2013). Phylogenetic analysis was performed with RAxML-NG (Kozlov et al. 2019) using the GTR + gamma model and 1000 bootstraps (Tineo et al. 2022). The tree was visualized with TreeDyn 198.3 at Phylogeny.fr (Dereeper et al. 2008).

## Results

### Genome organization and composition

**Figure 2.**
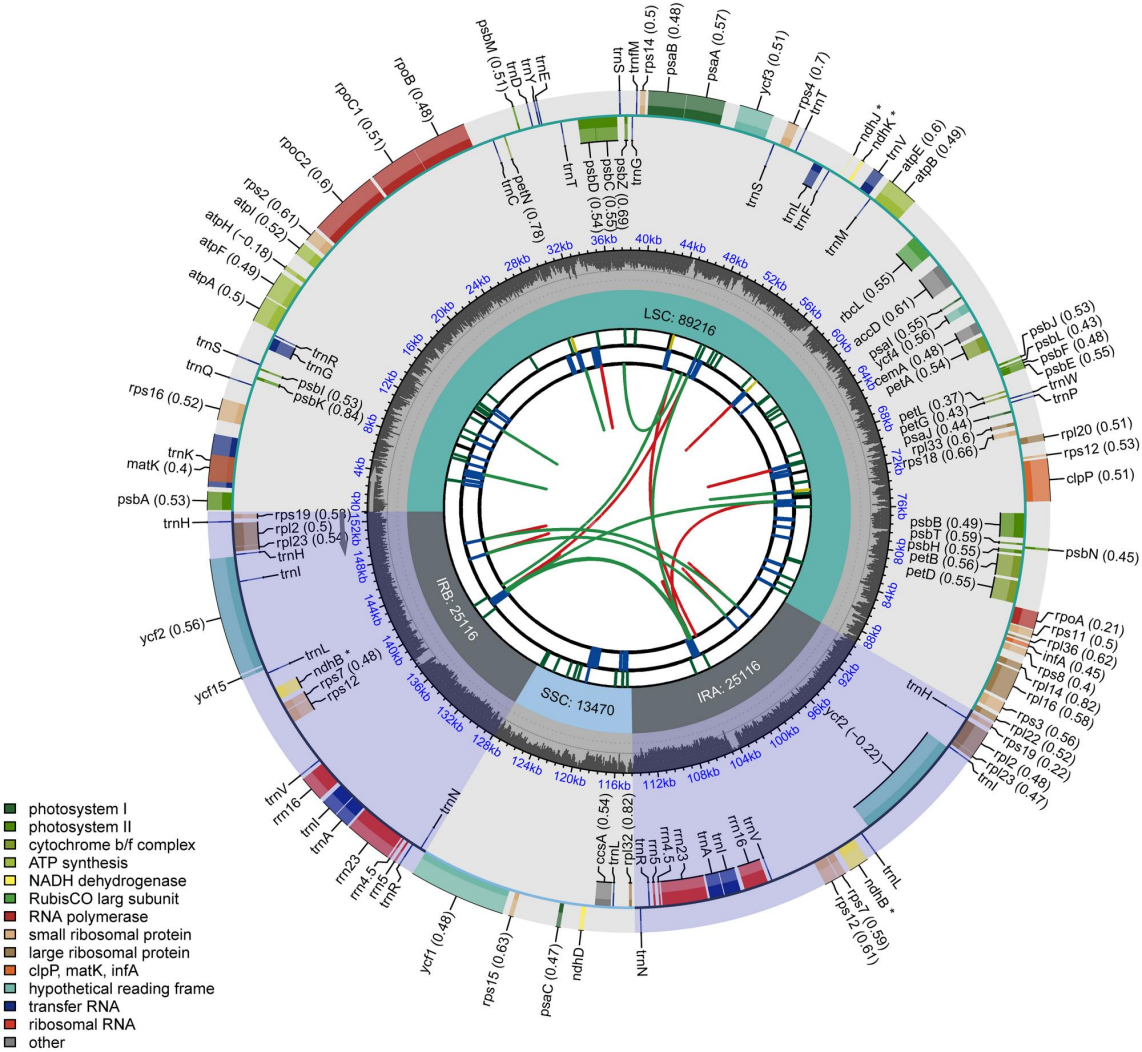
Schematic map of the general characteristics of the chloroplast genome of *Phragmipedium kovachii*. The map contains six tracks by default. From the center outward, the first track shows the scattered repeats connected with arcs. The second track shows the long tandem repeats as short bars. The third track shows the short tandem repeats or microsatellite sequences as short bars. The small single-copy (SSC), inverted repeat (IRa and IRb), and large single-copy (LSC) regions are shown in the fourth track. The GC content along the genome is represented in the fifth track. The genes are shown in the sixth track. Optional codon usage bias is shown in parentheses after the gene name. Genes are coded according to their functional classification. The transcription directions of the inner and outer genes are clockwise and counterclockwise, respectively. The functional classification of the genes is shown in the lower left corner.

The plastid genome of *P. kovachii* is 152,918 bp in length and contains 129 genes (Figure 2, Table S1), including a pair of inverted repeat regions (IRs) of 25,116 bp separated by a large single-copy (LSC) region of 89,216 bp and a small single-copy (SSC) region of 13,470 bp. The maximum, minimum, and average sequencing depths were 42,650, 23, and 538.6, respectively (Figure S1). This plastid genome is AT-rich (64.3%), comprising 26 ribosomal proteins, 39 tRNAs (*trn*A, *trn*G, *trn*N, *trn*H, and *trn*T occur in duplicate; *trn*R, *trn*V, and *trn*S occur in triplicate; and *trn*L and *trn*I occur in quadruplicate), eight rRNAs, 22 photosystem I and II genes, six *ycf* genes, seven cytochrome complexes (b6/f, f, b6, and c), six ATP synthases (CF0 and CF1), four RNA polymerases, and 11 other genes (*acc*D, *cem*A, *clp*P, *inf*A, *mat*K, and *ndh*B in duplicate, *ndh*D, *ndh*J, *ndh*K, and *rbc*L). Additionally, nine cis-splicing (*atp*F, *clp*P, *pet*B, *pet*D, *rpoC*1, *rpl*2, *rpl*16, *rps*16, and *ycf*3) and one trans-splicing (*rps12* with three exons) genes were identified (Figures S2 and S3). Fifty-four of the 129 genes were transcribed on the forward strand, and the remaining 75 were coded on the reverse strand.

**Figure 3.**
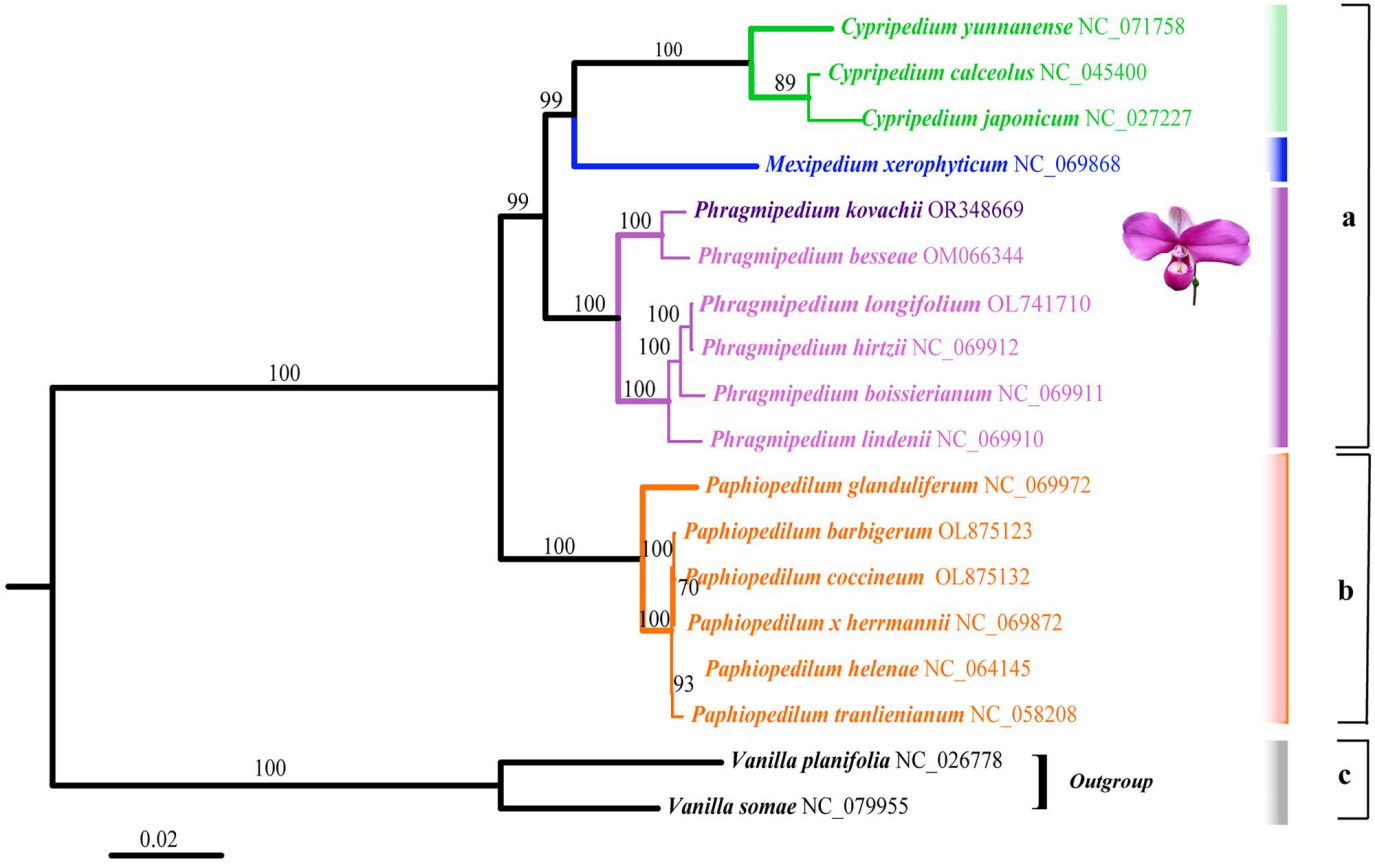
Maximum-likelihood phylogram of *Phragmipedium kovachii* (OR348669) and related genera within the subfamily Cypripedioideae. The following sequences were used: NC_071758 (Li et al. 2021), NC_045400 (Zhang et al. 2019), NC_027227 (Kim et al. 2015), NC_069868 (Hu et al. 2022), OR348669 (this study), OM066344 (Hu et al. 2022), OL741710 (Hu et al. 2022), NC_069912 (Hu et al. 2022), NC_069911 (Hu et al. 2022), NC_069910 (Hu et al. 2022), NC_069972 (Hu et al. 2022), OL875123 (Hu et al. 2022), OL875132 (Hu et al. 2022), NC_069872 (Hu et al. 2022), NC_064145 (Yen et al. 2022), NC_058208 (Yen et al. 2022), NC_026778 (Hua and Chen 2019), and NC_079955 (Cascales et al. 2023). Numbers along the branches are RAxML bootstrap support values based on 1500 replicates. The legend below represents the scale for nucleotide substitutions.

### Phylogenetic analysis

The presence of highly supported nodes in the phylogenetic analysis of plastid genome sequences from specimens within the subfamily Cypripedioideae confirmed that the genus *Phragmipedium* is sister to the clade comprising the genera *Cypripedium* and *Mexipedium* (Figure 3). This analysis also revealed that *P. kovachii* is a sister species of *P. besseae*.

## Discussion

Currently, 21 species of the genus *Phragmipedium* are accepted (Días-Morales et al. 2019), and the sixth complete plastid genome of this genus was assembled in the present study. The plastome of *P. kovachii* is highly conserved in length, content, and organization compared to that of other species assigned to *Phragmipedium* (Table 1). The plastid genome of *P. kovachii* was larger than that of the other members of *Phragmipedium* (Table 1).

**Table 1.** Plastid genome features among species of *Phragmipedium*, *Cypripedium*, *Mexipedium*, and *Paphiopedilum.*

Group		*Phragmipedium*						*Paphiopedilum*	*Cypripedium*	*Mexipedium*
Species		*P. besseae* (OM066344)	*P. boissierianum* (NC069911)	*P. hirtzii* (NC069912)	*P. kovachii* (OR348669*)*	*P. longifolium* (OL741710)	*P. lindenii* (NC069910)	*Pa. delenatii* (MK463585)	*C. japonicum* (NC027227)	*M. xerophyticum* (NC069868)
Total length (bp)		147,409	148,625	151,023	152,918	151,164	150,136	160,955	174,417	144,335
IR length (bp)		24,517	24,782	24,848	25,116	24,859	24,822	34,196	27,592	25,623
LSC length (bp)		86,096	87,331	88,252	89,216	88,378	87,234	89,869	97,322	82,348
SSC length (bp)		12,279	11,730	13, 075	13,470	13,068	13,258	2694	21,911	10,741
Total gene number		128	128	128	129	128	128	130	134	125
Coding sequence (CDS) number		74	74	74	74	74	74	77	85	74
rRNA number		8	8	8	8	8	8	8	8	8
tRNA number		38	38	38	39	38	38	39	38	38
Overall GC content (%)		37	36.4	36.1	35.7	36.1	36.1	35.6	35	37
GC content of IR (%)		43	42.8	42.8	42.1	42.8	42.8	39.3	43	43
GC content of LSC (%)		3. 4	33.9	33.5	35.7	33.4	33.5	33	32	3. 4
GC content of SSC (%)		29	28	28.2	28.3	28.2	28.1	28.5	26	28
Genes ndh	Presence	*ndh*J, *ndh*K, *ndh*B(x2), *ndh*D,	*ndh*J, *ndh*K, *ndh*B(x2), *ndh*D	*ndh*J, *ndh*K, *ndh*B (x2), *ndh*D	*ndh*B (x2), *ndh*D, *ndh*J, *ndh*K	*ndh*J, *ndh*K, *ndh*B(x2), *ndh*D	*ndh*J, *ndh*K, *ndh*B(x2), *ndh*D	*ndh*B(x2), *ndh*J, *ndh*K, *ndh*C, *ndh*D	*ndh*J, *ndh*K, *ndh*C, *ndh*B (x2), *ndh*F, *ndh*D, *ndh*E, *ndh*G, *ndh*I, *ndh*A, *ndh*H	*ndh*B(x2), *ndh*D
	Absence	*ndh*E, *ndh*I, *ndh*A, *ndh*H	*ndh*E, *ndh*I, *ndh*A, *ndh*H	*ndh*E, *ndh*I, *ndh*A, *ndh*H	*ndh*E, *ndh*I, *ndh*A, *ndh*H	*ndh*E, *ndh*I, *ndh*A, *ndh*H	*ndh*E, *ndh*I, *ndh*A, *ndh*H	*ndh*E, *ndh*I, *ndh*A, *ndh*H		*ndh*E, *ndh*I, *ndh*A, *ndh*H

*P. kovachii* lacks the following genes: *ndh*E, *ndh*I, *ndh*A, and *ndh*H. *ndh* genes are absent in *Paphiopedilum helenae* and very common in other species of *Phragmipedium*, *Mexipedium*, and *Paphiopedilum.* However, a greater number of *ndh* genes were reported in species of the genus *Cypripedium* (Lin et al. 2015). This difference is probably due to the putative transfer of all the *ndh* loci to the nucleus in the common ancestor of Cypripedioideae, which might explain the serial loss of the *ndh* genes in multiple orchid lineages (Kim et al. 2015). The plastid genome of *P. kovachii*, based on p-distance, genetically diverged from *P. besseae* (0.39%), *P. boissierianum* (1.02%), *P. hirtzii* (0.96%), *P. lindenii* (1.12%), and *P. longifolium* (0.96%) (Figure S4). This finding suggested that *P. kovachii* is highly similar to its sister species, *P. besseae.* In summary, this study provides a helpful framework for clarifying the phylogenetic connections of the genus *Phragmipedium* as well as a useful resource for conservation.

## Supplementary Material

Draft Plastome Pkovachii DEB04 Table supp.docx

Figure S3 Transsplicing gene.jpg

Figure S1 Sequenced deeph.jpg

Figure S2 Cissplicing genes.jpg

Figure S4 Divergenece Heapmap.jpg

## Data Availability

The genome sequence data that support the findings of this study are openly available from GenBank of the NCBI at https://www.ncbi.nlm.nih.gov/ under accession number OR348669. The associated BioProject, BioSample, and SRA numbers are PRJNA1076912, SAMN39956556, and SRR27984807, respectively.
