# Supplementary material for: High-throughput sequencing yields the complete plastid genome of the endemic species Phragmipedium kovachii (Orchidaceae) from northeastern Peru
Source: Mitochondrial DNA B Resour. 2024 Sep 2;9(9):1175–80. doi: 10.1080/23802359.2024.2397979 (PMC11378673; doi:10.1080/23802359.2024.2397979)
Supplement: Draft Plastome Pkovachii DEB04 Table supp.docx [file TMDN_A_2397979_SM1881.docx]

**High-throughput sequencing yields the complete plastid genome of the endemic species *Phragmipedium kovachii* (Orchidaceae) from northeastern Peru**

Jois V. Carrion^a^, Jhordy Perez^a^, Daniel Tineo^a^, Martha S. Calderon^a,b^, Ligia Garcia^a^, Manuel Oliva^a^, Oscar Gamarra^b^, Danilo E. Bustamante^a,b, *^

^a^Instituto de Investigación para el Desarrollo Sustentable de Ceja de Selva (INDES-CES), Universidad Nacional Toribio Rodríguez de Mendoza, Chachapoyas, Amazonas, Peru

^b^Instituto de Investigación en Ingeniería Ambiental (IIIA), Facultad de Ingeniería Civil y Ambiental (FICIAM), Universidad Nacional Toribio Rodríguez de Mendoza, Chachapoyas, Amazonas, Peru

**Table S1.** List of annotated genes in the plastid genome of *Phragmipedium kovachii.*

| **Gene classification** | | **Name of the genes** | **Total** |
| --- | --- | --- | --- |
| RNA genes | ribosomal RNA | *rrn*4.5(×2), *rrn*5(×2), *rrn*16(×2), *rrn*23(×2) | 8 |
|  | Transfer RNA | *trn*A_UGC (×2), *trn*C_GCA, *trn*D_GUC, *trn*E_UUC, *trn*F_GAA, *trn*fM_CAU, *trn*G_GCC, *trn*G_UCC, trnH-CAT, *trn*H_GUG(x2), *trn*I_CAU(×2), *trn*I_GAU(×2), *trn*K_UUU, *trn*L_CAA(×2), *trn*L_UAA, *trn*L_UAG, *trn*M_CAU, *trn*N_GUU(x2), *trn*P_UGG, *trn*Q_UUG, *trn*R_ACG(x2), *trn*R_UCU, *trn*S_GCU, trnS_UGA, *trn*S_GGA, *trn*T_GGU, *trn*T_UGU, trnV-GAC(x2), *trn*V_UAC, *trn*W_CCA, *trn*Y_GUA | 39 |
| Protein-coding genes | Photosystem I | *psa*A, *psa*B, ycf3, psaI, ycf4, psaC, *psa*J | 7 |
|  | Photosystem II | *psb*A, *psb*B, *psb*C, *psb*D, *psb*E, *psb*F, *psb*H, *psb*I, *psb*J, *psb*K, *psb*L, *psb*M, *psb*N, *psb*T, *psb*Z | 15 |
|  | cytochrome | *petA, petB, petD, petG, petL, petN, ccsA* | 7 |
|  | ATP synthase | *atp*A, *atp*B, *atp*E, *atp*F, *atp*H, *atp*I | 6 |
|  | Rubisco | *rbc*L | 1 |
|  | Ribosomal proteins-small units | *rps*11, *rps*12(×2), *rps*14, *rps*15, *rps*16, *rps*18, *rps19*(×2), *rps*2, rps3, *rps*4, *rps*7(×2), *rps*8 | 15 |
|  | Ribosomal proteins: large units | *rpl14,* *rpl16,* *rpl2*(×), *rpl20*, *rpl22*, *rpl*23(×2), *rpl32*, *rpl33*, *rpl36* | 11 |
|  | RNA polymerase | *rpo*A, *rpo*B, *rpo*C1, *rpo*C2 | 4 |
|  | Miscellaneous | *acc*D, *cem*A, *clpP*, *inf*A, *mat*K | 5 |
|  | Hypothetical chloroplast reading frames (ycf) | *ycf*1, *ycf*2(×2), *ycf*3, *ycf*4*, ycf15* | 6 |
| Pseudogenes | Pseudogenes | *ndh*B(×2), *ndh*D, *ndh*J, *ndh*K, | 5 |
| **Total** | | | **129** |
