# Supplementary figures and images for: High-throughput sequencing yields the complete plastid genome of the endemic species Phragmipedium kovachii (Orchidaceae) from northeastern Peru
Source: Mitochondrial DNA B Resour. 2024 Sep 2;9(9):1175–80. doi: 10.1080/23802359.2024.2397979 (PMC11378673; doi:10.1080/23802359.2024.2397979)

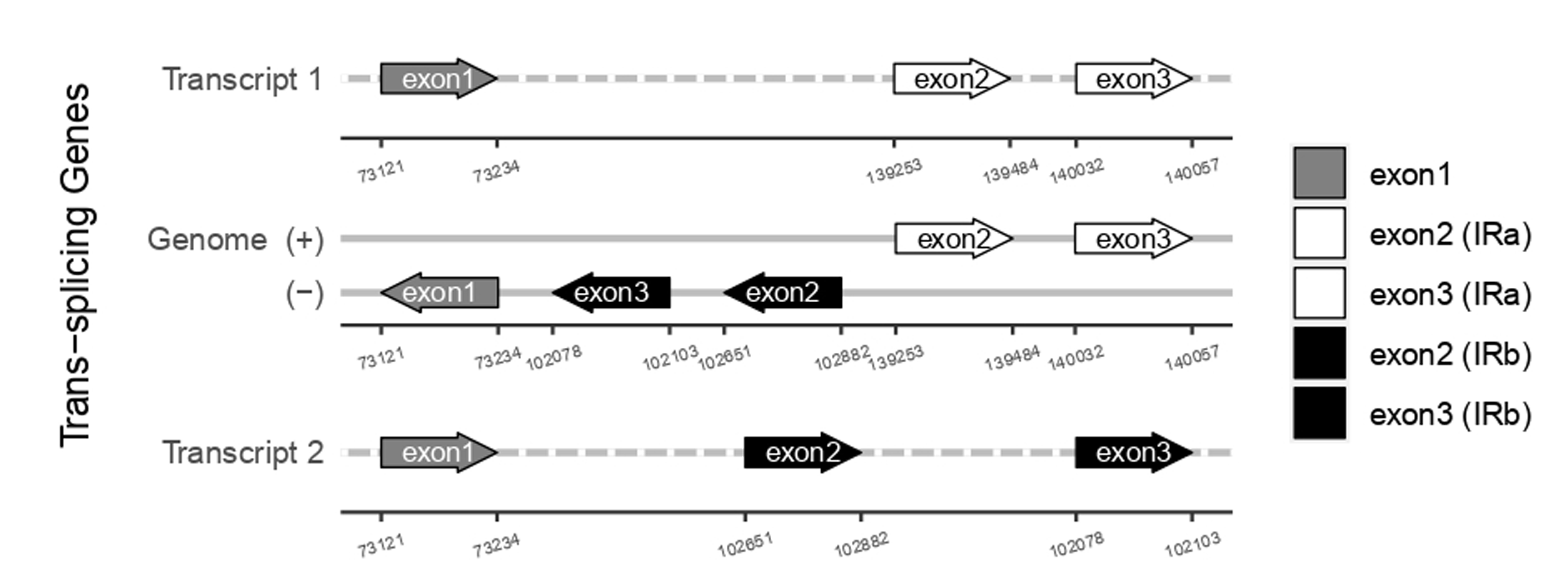

Supplement: Figure S3 Transsplicing gene.jpg [file TMDN_A_2397979_SM1880.jpg]

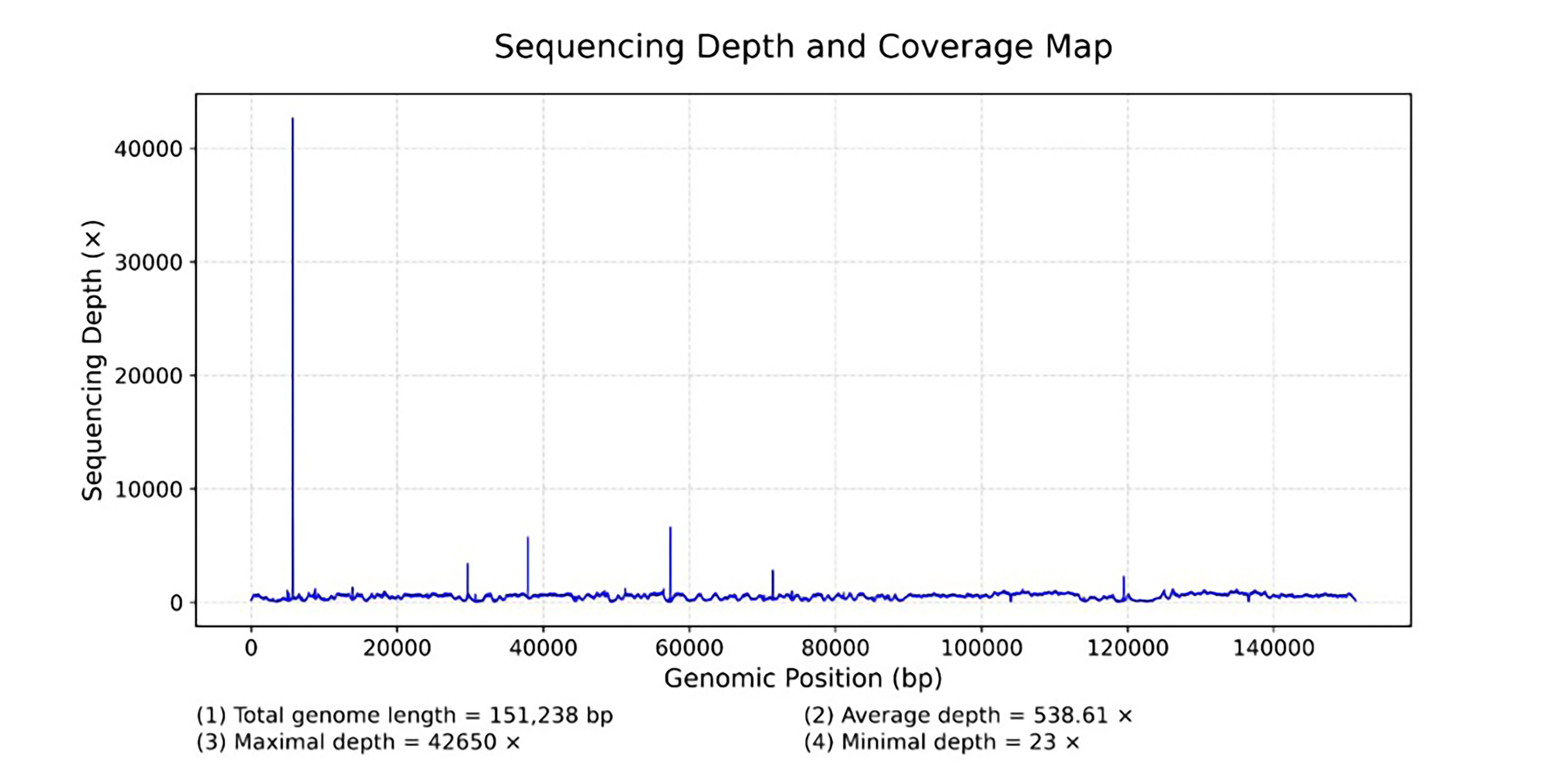

Supplement: Figure S1 Sequenced deeph.jpg [file TMDN_A_2397979_SM1879.jpg]

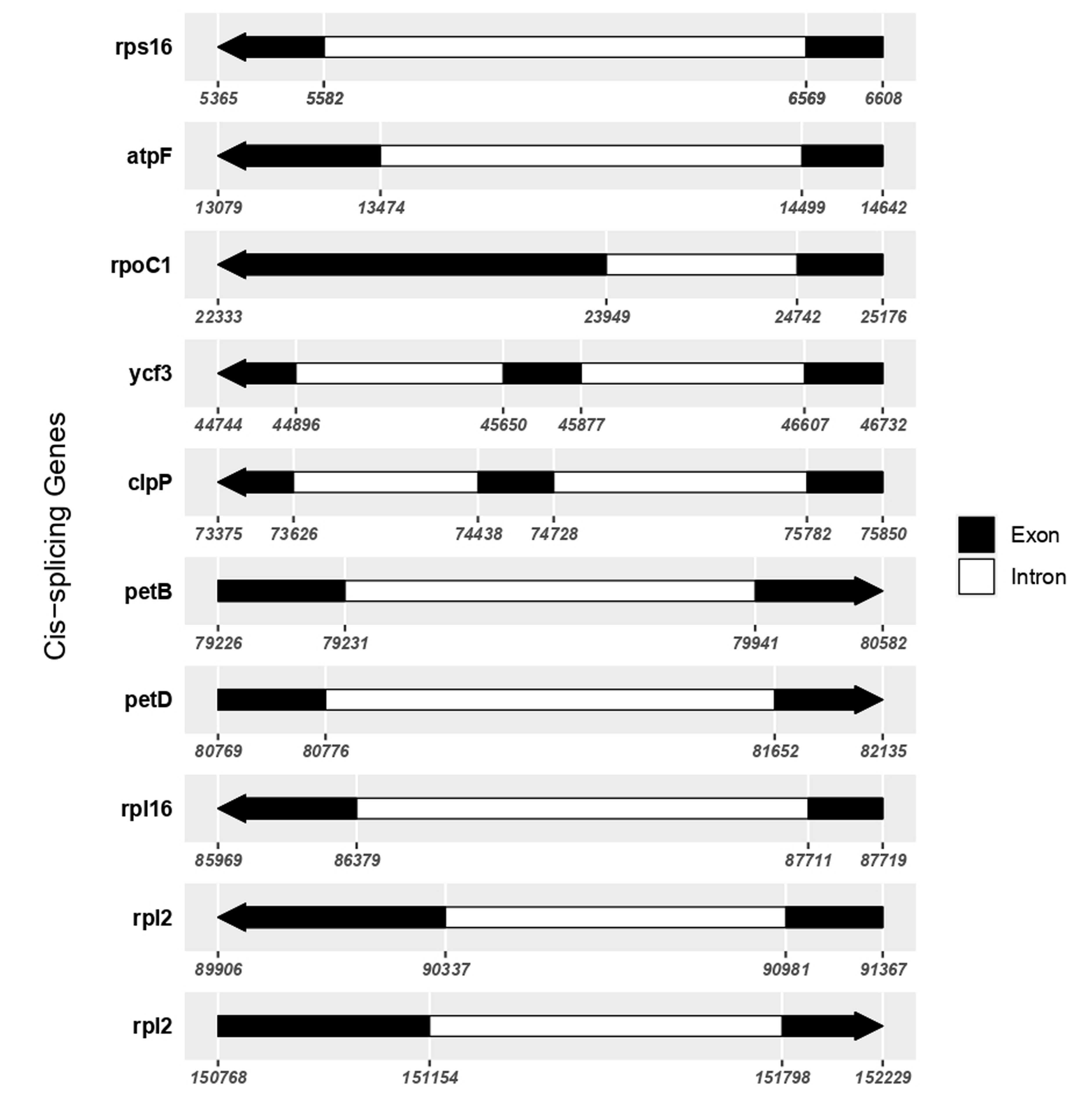

Supplement: Figure S2 Cissplicing genes.jpg [file TMDN_A_2397979_SM1878.jpg]

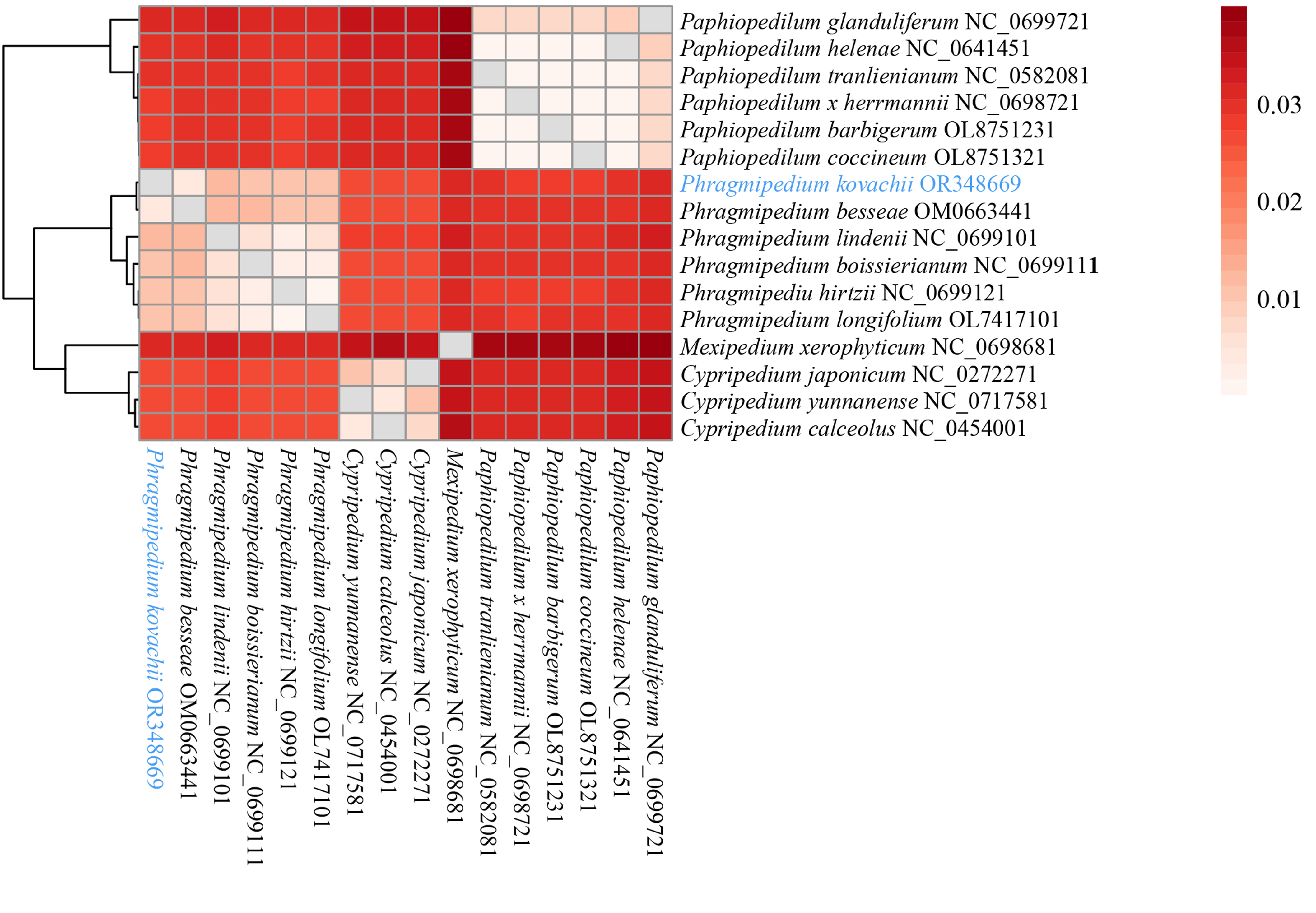

Supplement: Figure S4 Divergenece Heapmap.jpg [file TMDN_A_2397979_SM1877.jpg]
